# Robotic female artificial urinary sphincter implantation vs. male artificial urinary sphincter implantation for non-neurogenic stress urinary incontinence

**DOI:** 10.1007/s00345-024-05238-0

**Published:** 2024-10-03

**Authors:** Alexandre Dubois, Valentine Lethuillier, Claire Richard, Camille Haudebert, Imad Bentellis, Mehdi EL-Akri, Lucas Freton, Andrea Manunta, Juliette Hascoet, Benoit Peyronnet

**Affiliations:** 1 Department of Urology, University Hospital of Rennes, Rennes, France; 2 Department of Urology, Polyclinique Saint Jean, Cagnes-sur-Mer, France; 3 Department of Urology, Clinique Mutualiste La Sagesse, Rennes, France

**Keywords:** Artificial urinary sphincter, Stress urinary incontinence, Intrinsic sphincter deficiency, Male, Female, Comparison

## Abstract

**Purpose:**

Previous studies suggested better functional outcomes and longer device survival for female artificial urinary sphincter (AUS) implantation compared to male AUS implantation. We hypothesized that the adoption of robotic approaches for female implantation might have influenced these comparisons. This study aimed to compare the outcomes of robotic female AUS and male AUS implantation for non-neurogenic stress urinary incontinence (SUI).

**Methods:**

We retrospectively reviewed charts of male patients who had AUS implantation and female patients who underwent robotic AUS implantation for non-neurogenic SUI between 2010 and 2022 at a single center. Prior AUS implantations were exclusion criteria. The primary endpoint was continence status at 3 months, categorized as complete resolution of SUI (0 pad), improved SUI (1pad), or unchanged SUI (>1pad).

**Results:**

After excluding 79 patients, 171 were included: 70 women and 101 men. Operative time was shorter in males (126.9 vs. 165.5 min; *p* < 0.0001). Postoperative complication rates were similar (17.3% vs. 22.9%; *p* = 0.38). Continence status at 3 months and last follow-up favored females. The ICIQ-SF decrease at 3 months was greater in females (-7.2 vs. -4.6; *p* < 0.001). The 5-year estimated explantation-free survival was similar (78.6% vs. 73.7%; *p* = 0.94) as was the revision-free survival (67.4% vs. 61.7%; *p* = 0.89). Multivariate analysis showed that female gender was associated with better continence at last follow-up (OR = 4.3; *p* = 0.03).

**Conclusion:**

Robotic female AUS implantation is associated with better functional outcomes than male AUS implantation, with similar morbidity and survival rates.

## Introduction

The first artificial urinary sphincter (AUS) device was described in 1973 by F.B. Scott [1]. After a few modifications to the primary device, the AMS800 by Boston Scientific© is currently the most commonly used AUS.

It is mainly used to treat both neurogenic and non-neurogenic male stress urinary incontinence (SUI). It is also used in some countries to treat female SUI due to intrinsic sphincter deficiency (ISD).

The EAU guidelines do not recommend the AUS as a first-line treatment for female patients due to insufficient high-quality data and a quite high rate of complications [2]. However, AUS implantation in women is becoming increasingly prevalent in some countries [3], presumably due to the evolution of the surgical technique and the increasing adoption of the robot-assisted approach.

Two series have suggested better functional outcomes and longer device survival for female AUS implantation compared to male AUS implantation [4, 5]. Petero et al. found an higher fully dry rate and a longer device duration for women while Cotte et al. found a lower revision rate for women and a higher explantation rate for men. Preliminary results of robot-assisted compared with open AUS implantation in female patients have suggested a decrease in complication rates, length of hospital stay, and blood loss [6].

We hypothesize that the rapidly spreading robotic approach for female implantation may have changed how female AUS compares to male AUS. The aim of this study was to compare the outcomes of robotic female AUS vs. male AUS implantation for non-neurogenic SUI.

## Methods

### Study design

After institutional board review approval (CNIL number: 2234449v0), the data of all female and male patients who underwent a robotic AUS implantation at a single academic center between January 2010 and December 2022 were collected prospectively (female patients) or retrospectively (male patients). All the implantations were performed by three surgeons in their learning curves over the study period. The database was analyzed retrospectively for the purpose of the present study.

Prior AUS implantation and neurogenic SUI were the only exclusion criteria. The following baseline characteristics were recorded in a dedicated computerized dataset for all patients: age at the time of AUS implantation, body mass index (BMI), ASA score, etiology of incontinence (neurogenic vs. non-neurogenic), history of radiotherapy, history of previous anti-incontinence surgery, number of pads per day, type of pad, presence of urgency, maximum free urinary flow (Qmax), post-void residual volume (PVR). All patients had to fill out the International Consultation on Incontinence Questionnaire – Short Form (ICIQ-SF) and Urinary Symptom Profile questionnaire (USP) prior to surgery and at any follow-up clinics as part of the institution’s routine practice. Unlike for female implantations, male patients did not have a systematic urodynamic assessment prior to surgery.

### Patient’s selection

For male patient, AUS was offered as an option to all patients presenting a positive cough stress test. 

AUS was offered to all female patients with SUI due to ISD defined as a positive cough stress test with a poorly mobile or fixed urethra on physical examination. Urodynamic was performed in all of these female patients and a low maximum urethral closure pressure (MUP) was deemed as a co argument of ISD.

### AUS implantation and activation

All female AUS were implanted using an anterior transperitoneal robot assisted approach according to the technique previously described [7] except for two implanted using a preperitoneal robot-assisted approach. The sphincter cuff was placed around the bladder neck in all cases.

For male AUS, the sphincter cuff was placed around the bulbar urethra. The surgical approach was either perineal or penoscrotal depending on the surgeon’s preference.

The urethral catheter was removed in the operative room or on day 1 postoperatively, except in cases of bladder injury where it was kept for 10 to 14 days. The AUS was activated at six weeks for all patients and then the patient was seen at 3 months and then yearly and in case of any intercurrent event.

### Outcomes of interest

The primary endpoint of the present study was the continence status at 3 months categorized as: complete resolution of SUI (0 pad), improved SUI (1 pad a day) or unchanged SUI (> 1 pad a day).

The secondary endpoints were (I) continence status at last follow-up (II) post-operative complications (III) major postoperative complications defined as a Clavien Dindo Grade of 3 or higher (IV) explantation free survival of the device (V) revision free survival of the device (VI) Other perioperative outcomes: mean operative time, estimated blood loss, cuff size, length of hospital stay.

The postoperative complications were recorded and graded using the Clavien-Dindo classification [8].

The last follow-up was defined as the most recent date on which information was gathered, either during an outpatient clinic or by phone call to update the patient’s data.

Explantation was performed when cuff or pump erosion occurred or in cases of device infection. It was also offered in rare cases for difficulties in manipulating the pump that remained unsolved despite repeat hospitalizations for patients’ education. A revision of the device was performed for mechanical or non-mechanical failure. In rare occasions, it was used to relocate the pump after difficulties manipulating it or because of pain in seated-position.

### Statistical analysis

Means and standard deviations were reported for continuous variables, medians and ranges for categorical variables, and proportions for nominal variables. Comparisons between groups were performed using the χ^2^ test or Fisher’s exact test for discrete variables, and the Mann-Whitney test for continuous variables as appropriate.

We performed multivariate logistic regression analysis to assess the predictive factors of continence at the last follow up. The probabilities of revision-free and explantation-free survivals were estimated using the Kaplan-Meier method.

Patients without any event (revision or explantation) during the study period were censored at the date of the last follow-up. Statistical analyses were performed using JMP v.12.0 software (SAS Institute Inc., Cary, NC, USA).

All tests were two-sided with *p* < 0.05 as a threshold to define statistical significance.

## Results

### Patients’ characteristics

After the exclusion of 79 patients (60 men and 19 women), 171 patients (101 men and 70 women) were included. The two populations differed significantly. The median age was higher in the male population (73 vs. 65.5; *p* < 0.0001) and men had a lower body mass index (BMI) (26.1 vs. 28.1; *p* < 0.0001). Antiplatelet agents’ intake was more common in male patients (28.3% vs. 5.7%; *p* < 0.0001) and so was history of previous pelvic radiotherapy (42%vs. 2%; *p* < 0.0001). Conversely, an history of previous midurethral slings was more prevalent in the female group (75.7% vs. 11.8%; *p* < 0.0001).

Regarding urodynamic parameters, detrusor overactivity was more prevalent in men (27% vs. 14%; *p* = 0.008) and cystometric capacity was lower in men. (284.4 vs. 442.9; *p* = 0.01) (See Table 1).

**Table 1:** Patients’ characteristics

	Male AUS *N* = 101	Robotic female AUS *N* = 70	*p*-value
Median age (years)	73 (range:54–92)	66.5 (range:38–85)	< 0.0001
Mean Body Mass Index (kg/m2)	26.1 (+/- 3.1)	28.1 (+/- 5.5)	< 0.0001
Anticoagulant intake	12 (12.2%)	6 (8.7%)	0.46
Antiaggregant intake	28 (28.3%)	4 (5.7%)	< 0.0001
History of radiation therapy	42 (42%)	2 (2.9%)	< 0.0001
History of synthetic sling	12 (11.8%)	53 (75.7%)	< 0.0001
History of Adjustable Continence Therapy periurethral balloon	9 (8.9%)	8 (11.6%)	0.57
ASA Score			
1	12 (13.8%)	20 (28.6%)	0.05
2	60 (69%)	43 (61.4%)	
3	15 (17.2%)	7 (10%)	
Detrusor overactivity on preoperative urodynamic	27 (41.5%)	14 (20.3%)	0.008
Mean Maximum cystometric capacity on preoperative urodynamic (ml)	384.4 (+/- 123.7)	442.9 (+/- 114.6)	0.01

### Perioperative outcomes

The mean operative time was shorter for male AUS implantation (126.9 vs. 165.5 min; *p* = 0.0009). The median cuff size differed significantly between male and female patients (median cuff size: 45 mm vs. 75 mm; *p* < 0.0001). The rates of postoperative complications (17.3% vs. 22.9%) and major postoperative complications (8% vs. 7.2%) did not differ significantly between the two groups. (*p* = 0.38 And *p* = 0.99 respectively).Major postoperative complications in women include 3 vaginal erosions ; 2 urethrovaginal fistulas with cuff exposure ; 1 abdominal wall abscess requiring surgical intervention ; 1 infection and one unknown cause. Except the abdominal wall abscess, all these complications lead to device removal. In the male population, major postoperative complications were represented by 1 hematoma ; 1 acute urinary retention and 3 unknown causes.

### Functional outcomes

The continence status at 3 months was better in the female AUS group with 48 female patients vs. 46 male patients reporting complete continence respectively (68.6% vs. 50.5%; *p* = 0.048) (See Table 2).

**Table 2:** Peri et post-operative outcomes

	Male AUS *N* = 101	Robotic female AUS *N* = 70	*p*-value
**Mean operative time (min)**	126.9 (+/- 92.7)	165.5 (+/- 54.9)	0.0009
**Median AUS cuff size (mm)**	45 (35–55)	75 (70–90)	< 0.0001
**Postoperative complications**	17 (17.3%)	16 (22.9%)	0.38
**Major postoperative complications (Clavien grade 3 or higher)**	8 (8%)	5 (7.2%)	0.99
**Median length of hospital stay (days)**	2 (0–7)	2 (0–8)	0.45
**Median number of pads/24 h**			
Preoperatively	4 (1–16)	4 (1–20)	0.81
3 months postoperatively	1 (0–5)	0 (0–4)	0.004
Change	− 3	− 4	< 0.001
**Mean ICIQ SF (/21)**			
Preoperatively	17.3 (+/- 4)	16.4 (+/- 3.7)	0.30
3 months postoperatively	7.1 (+/- 4.7)	3.2 (+/-5.5)	0.002
Change	-10.2	− 13.2	< 0.001
**Mean ICIQ SF- qol (/10)**			
Preoperatively	7.7 (+/- 2.9)	8.5 (+/- 1.8)	0.36
3 months postoperatively	3.1 (+/- 3.8)	1.3 (+/-2.8)	0.048
Change	-4.6	− 7.2	< 0.001
**Mean USP SUI subscore (/9)**			
Preoperatively	7.8 (+/- 2)	7.5 (+/- 2.5)	0.69
3 months postoperatively	2.4 (+/- 3)	0.8 (+/-1.9)	0.005
Change	-5.4	− 6.7	< 0.001
**Continence status at 3 months**			
Complete continence	46 (50.5%)	48 (68.6%)	0.048
Improved continence	26 (28.6%)	15 (21.4%)	
Unchanged continence	29 (20.9%)	7 (10%)	
**Continence status at last follow-up**			
Complete continence (no pad)	49 (53.3%)	50 (72.5%)	0.01
Improved (1 pad) or unchanged continence (1 pad or more)	43 (46.7%)	19 (27.5%)	
**Median follow-up (months)**	42 (3-153)	18 (3-101)	0.0004

Functional outcomes at last follow-up still favored female patients with 50 female patients describing a complete continence versus 49 men (72 vs. 53.3% ; *p* = 0.01). The ICIQ-SF decrease at 3 months was significantly greater in the female group (-7.2 vs. -4.6 ; *p* < 0.001). We observed the same results for the USP Stress Urinary Incontinence sub score. (-5.4 vs. -6.7 ; *p* < 0.001)

### Predictive factors

In multivariate analysis, the only predictive factor of complete continence at last follow-up was female gender with an odds ratio (OR) of 4.23 [1.11–18.13] ; *p* = 0.03 (See Table 3). An history of midurethral sling, radiation therapy, BMI, the number of pads per day and preoperative detrusor overactivity were not predictive factors of complete continence at the last follow-up.

**Table 3:** Predictive factors of complete continence at last follow-up

	Odds-ratio [CI-95%]	*p*-value
History of midurethral sling	0.67 [0.18–2.18]	0.51
Female gender	4.23 [1.11–18.13]	0.03
Radiation therapy	0.95 [0.25–3.60]	0.94
Age	0.36 [0.09–2.76]	0.42
Detrusor overactivity	0.44 [0.16–1.23]	0.12
BMI	0.95 [0.09–2.76]	0.98
Number of pads per 24 H	0.08 [0.02–12.23]	0.13
Maximum cystometric capacity	0.71 [0.09–24.3]	0.81

### Estimated survival rates

**Fig. 1:**
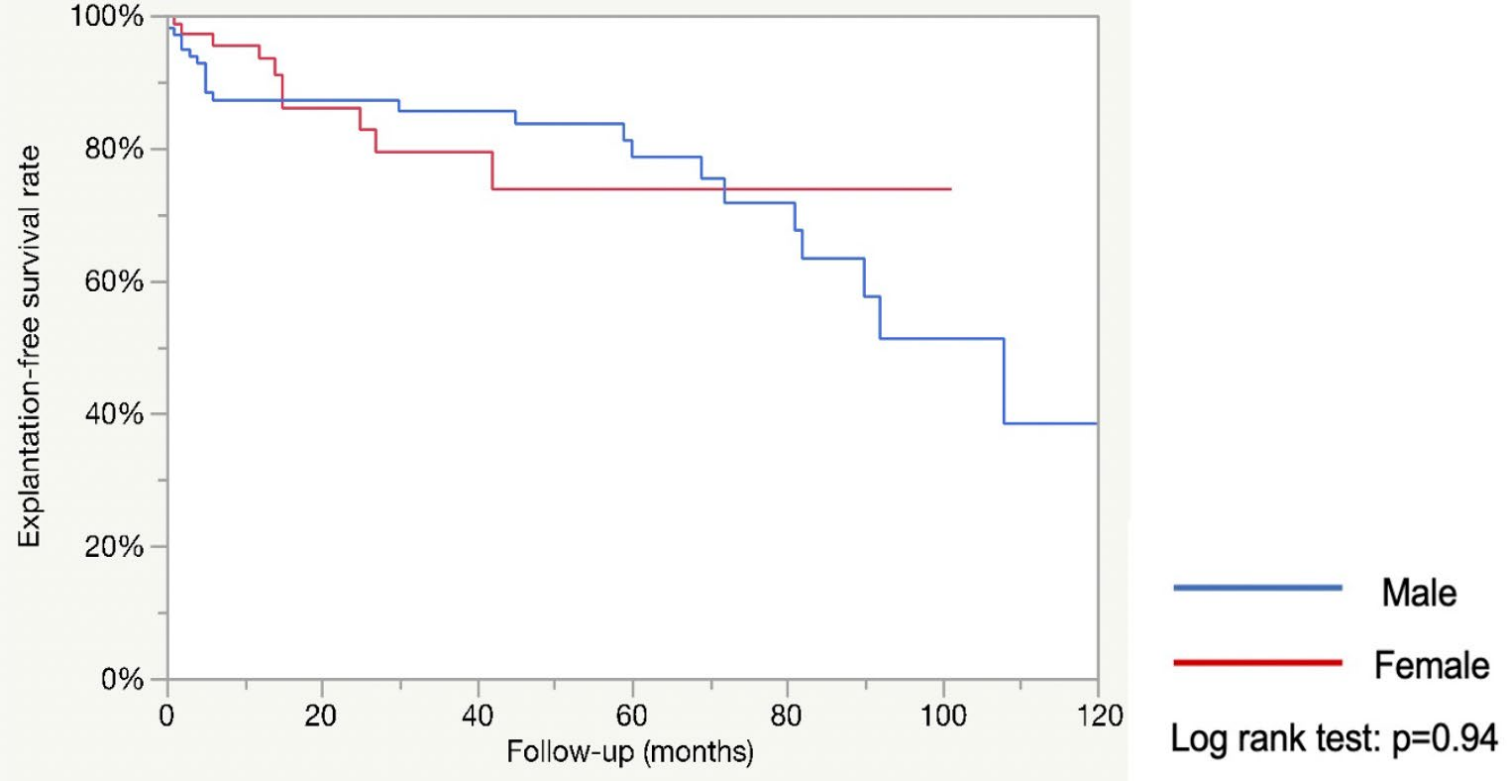
Estimated explantation-free survival rate

**Fig. 2:**
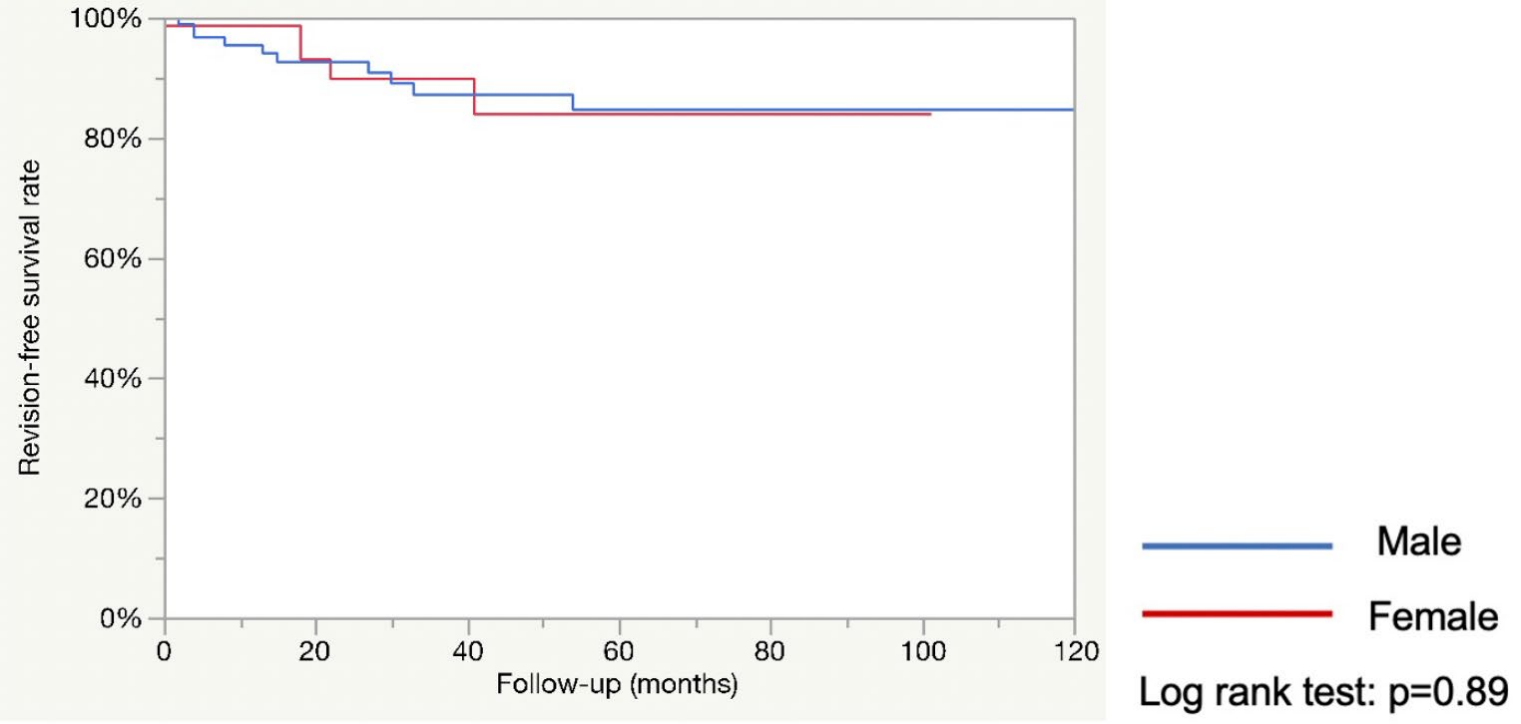
Estimated revision-free survival rate

The median follow-up was much longer in the male population. (42 vs. 18 months ; *p* < 0.0004). The 5-year estimated explantation-free survival was similar in both groups (78.6% vs. 73.7% %; *p* = 0.94: see Fig. 1) as was the 5-year estimated revision-free survival (67.4% vs. 61.7%; *p* = 0.89; see Fig. 2).

## Discussion

The present study provides a new comparison between female AUS and male AUS implantation taking into account the rapidly spreading robotic approach for female implantation. Despite differences (populations, cuff implantation sites, and surgical indications), robotic female AUS was associated with better functional outcomes than bulbar male AUS implantation in terms of continence and patients reported outcomes. Morbidity and survival rates are similar between the two population. It adds another argument supporting the use of AUS to treat female stress urinary incontinence due to intrinsic sphincter deficiency.

Our two populations differ in many ways. The higher incidence of radiotherapy in men may be due to the fact that stress urinary incontinence occurs mainly after prostate cancer treatment. Stereotactic radiotherapy is frequently used for treatment or retreatment in this area. This explains the higher proportion of detrusor overactivity and lower cystometric capacity in this population, as radiation therapy alters tissues and induces bladder wall fibrosis [9]. Radiation on the urethra could impact the post-operative pad-free rate in the male population by altering its compliance, potentially leading to device failure. AUS implantation in women with a history of radiotherapy is rare despite pelvic radiotherapy is a frequent treatment for gynecological cancers. This rarity may due to pelvic radiotherapy in women having a greater impact on the bladder than in men, given the area of irradiation involved [10–12]. As the bladder may be the predominant pathophysiological determinant of urinary incontinence, indications for outlet procedures are less prevalent in female patients who underwent pelvic radiotherapy.

In terms of consequences, the primary risks of men AUS implantation after pelvic radiotherapy are failure due to coexistent or persistent bladder dysfunction [13, 14] and an increased risk of cuff erosion or device infection [15]. However, explantation of a bulbar AUS in a male patient carries minimal risk of long-term sequelae [16]. In female AUS implantation, a history of pelvic radiotherapy has long been considered a major risk factor for AUS failure [17]. Unlike in men, where the cuff is placed around the bulbar urethra, the placement of the cuff at the bladder neck in woman can lead to more severe consequences, such as erosion or pubic bone infection [18]. This may explain why surgeons are more reluctant to use AUS in irradiated female patients, leading to a potential selection bias to bear in mind when analyzing the findings of the present study.

Additionally, fewer options exist for irradiated men as Adjustable Continence therapy (ACT) balloons and sub urethral slings are not recommended for this population [19]. Conversely, there are other alternatives available for the treatment of SUI due to ISD in irradiated women, such as peri-urethral bulking agents, autologous pubovaginal slings, and proACT balloons. This may partly explain the higher proportion of irradiated men compared to women in our study. Further studies with a larger proportion of female patients with a history of pelvic radiotherapy may be needed to determine if our results remain similar in this subgroup.

Another significant difference between the two groups at baseline was the higher proportion of patients with a history of previous anti-incontinence surgery in the female group. This may due to the differing physiopathology of SUI between men and women, with urethral hypermobility being a major determinant of female SUI [20]. As a result, AUS is typically offered to female patients who have failed one or more previous anti incontinence surgeries, such as midurethral sling or colposuspension. Hence AUS is mostly offered to female patients who have failed one or multiple previous surgical procedures [21, 22] as emphasized in most of existing guidelines [2].

Although pelvic radiotherapy and a history of previous anti incontinence surgery can be confounding factors as they alter tissue and make the procedure more complex, our multivariate analysis did not identify these factors as predictors of continence at last follow-up. We included patients with a history of pelvic radiotherapy and female patients with prior anti-incontinence surgery in our study design to reflect current practice. However, we chose not to include neurological patients, as they represent a specific and complex population, most of whom use intermittent self-catheterization (ISC). The impact of ISC on bladder neck versus bulbar urethra AUS outcomes may differ significantly, as the thicker bladder neck wall is believed to be less prone to cuff erosion from repeat catheterization [23]. Prior AUS implantation was an exclusion criterion, as the effects of previous AUS explantations remains elusive and patients eligible for a secondary or tertiary sphincter implantation have complex medical history with many confounding factors.

Our findings of better functional outcomes for female versus male AUS patients are consistent with previous reports. These results may be due to the cuff’s bladder neck location where a larger cuff size and thicker tissue provide an advantage compared to the bulbar urethra. Moreover, this location may allow abdominal pressure to be transmitted to the cuff, enhancing continence – an effect that may not occur with a bulbar urethra cuff. Pelvic radiotherapy may also play a role, as vessel inflammation, perivascular fibrosis, and edema lead to wall ischemia and tissue fibrosis. This leads to the loss of smooth muscle cells and the infiltration of tissues by collagen [11], altering the urethra’s physiological capacities. This could explain the partial effectiveness of AUS cuff in irradiated patients which was more prevalent in the male group.

The present study has several limitations that should be acknowledged. Our primary endpoint is debatable as it is subjective and does not reflect patients’ overall quality of life. For example, using more than one pad per day does not necessarily reflect any change in continence after AUS implantation. The single tertiary center where the study was conducted represents numerous inherent biases, as the cases may be more complex than those typically seen at other institutions. Finally, a key limitation is the shorter median follow-up time for the female population, which could result in an underestimation of explantation and revision rates. But it did not affect our primary endpoint which is continence at 3 months post-op. A longer follow-up would be valuable to confirm that these results are maintained over time. In addition, the rapidly spreading robotic approach for female implantation will most likely provide us larger populations and new data to confirm the present findings.

## Conclusion

Although baseline populations’ characteristics differ, robotic female AUS was associated with better functional outcomes than bulbar urethra male AUS implantation with similar morbidity and device survival rates. Even if further data with longer follow-up is needed, there seems to be no reason to offer AUS only to male patients in current times.

## Data Availability

This study was approved by the CNIL (Comité National Informatique et Liberté, CNIL : 2234449v0) and data is provided within the manuscript or supplementary information files on demand.

## Abbreviations

AUS: Artificial Urinary Sphincter
SUI: Stress Urinary Incontinence
ISD: Intrinsic Sphincter Deficiency
ICIQ-SF: International Consultation on Incontinence Questionnaire – Short Form
USP score: Urinary Symptom Profile score
ISC: intermittent self-catheterization
ACT or proACT balloons: Adjustable Continence Therapy balloons
